# Television airings of U.S. federal COVID-19 public service announcements in 2020 were associated with market-level political orientation, not COVID-19 rates

**DOI:** 10.1371/journal.pone.0275595

**Published:** 2022-10-06

**Authors:** Sarah E. Gollust, Chris Frenier, Margaret Tait, Colleen Bogucki, Jeff Niederdeppe, Steven T. Moore, Laura Baum, Erika Franklin Fowler

**Affiliations:** 1 Division of Health Policy and Management, University of Minnesota School of Public Health, Minnesota, Minneapolis, United States of America; 2 Department of Health Policy and Management, Yale School of Public Health, United States of America; 3 Division of Health and Environment, Abt Associates, United States of America; 4 Department of Communication, Cornell University, Ithaca, New York, United States of America; 5 Cornell Jeb E. Brooks School of Public Policy, Cornell University, Ithaca, New York, United States of America; 6 Department of Government, Wesleyan University, Middletown, Connecticut, United States of America; 7 Wesleyan Media Project, Wesleyan University, Middletown, Connecticut United States of America; Universitat Luzern, SWITZERLAND

## Abstract

Televised public service announcements were one of the ways that the U.S. federal government distributed health information about the COVID-19 pandemic to Americans in 2020. However, little is known about the reach of these campaigns or the populations who might have been exposed to the information these ads conveyed. We conducted a descriptive analysis of federally-affiliated public service announcement airings to assess where they were aired and the market-level social and demographic characteristics associated with the airings. We found no correspondence between airings and COVID-19 incidence rates from March to December 2020, but we found a positive association between airings and the Democratic vote share of the market, adjusting for other market demographic characteristics. Our results suggest that PSAs may have contributed to divergent exposure to health information among the U.S. public during the first year of the COVID-19 pandemic.

## Introduction

The COVID-19 pandemic revealed major inadequacies in the United States’ health communication strategy, including inconsistencies across federal and state agencies and conflicting messaging [1, 2]. A less-examined communication approach that the federal government used in responding to the pandemic was coordinating health information campaigns, also known as (PSAs). PSAs are promotional campaigns for topics of broad community interest that typically aim to increase public awareness of a problem and/or shape public beliefs, attitudes, or behaviors [3, 4]. In some cases, most notably in the context of U.S. tobacco control and obesity prevention efforts, state and federal government agencies have purchased airtime for public interest-serving health and social PSAs [5, 6]. Many PSA campaigns, however, rely on donated airtime. While the federal government does not mandate broadcasters to air PSAs, television stations that air them can meet their Federal Communications Commission licensing requirement to “serve the public interest” [7].

On March 17, 2020, the non-profit organization Ad Council launched a national PSA campaign in collaboration with the White House, Centers for Disease Control and Prevention, U.S. Department of Health and Human Services, and television networks, to “provide critical and urgent messages to the American public” [8]. As indicated in the campaign’s press release, media companies committed to air these ads on TV, radio, social media, billboards, and digital media using donated time [8]. Although there have been a few journalistic accounts of the federal coronavirus PSA campaign, examining, for instance, the types of celebrities that the Trump administration tried to recruit to participate [9, 10], there has been very little scientific research examining the role of or impact of PSAs in informing the public about COVID-19.

There is evidence that large-scale PSA campaigns can accelerate changes in health behavior at the population level [11]. The main benefit of PSA campaigns lies in their ability to reach large segments of the population repeatedly with content that conveys clear and concise messages about the benefits of performing a behavior (or avoiding an unhealthy behavior). While the strongest evidence for benefit comes from a very large body of campaign evaluation related to tobacco prevention and control [12, 13], there is at least moderate evidence for benefit from campaigns focused on topics as varied as physical activity [14], nutrition [15], HIV prevention [16], and road safety [17]. Overall, PSA campaigns are more likely to be successful when they achieve high levels of exposure at the population level and when they are combined with other community-level interventions that support the targeted behavior [11, 16]. While paid PSA campaigns are often able to achieve higher levels of exposure than those that are left to the discretion of broadcasters themselves [5, 18], at least some PSA campaigns relying on donated airtime have also shown evidence of effects in shifting behavior and health outcomes [17].

While research such as that reviewed above has examined the content of past PSA campaigns and the relationship between ad exposure and behavioral outcomes, research on the overall reach and specific patterns of PSA distribution is fairly limited. The few studies that have examined PSA distribution have indicated that television is the most widely used medium for PSAs [4], but that PSAs comprise a very small proportion of overall broadcast airtime. A 2008 study, for instance, found that broadcast and cable networks spent only 0.5% of their airtime on PSAs [7]. In addition, research suggests that competition for airtime of PSAs is quite heavy, and PSA sponsors that rely on donated time (which are the vast majority of PSAs aired from public and non-profit sponsors) are often relegated to less desirable times than are paid ads, such as overnight [3, 7, 17]. Further, past research has described substantial, and often unexplained, geographic variation in the distribution of PSAs aired across media markets. For instance, one study documented “extreme variation” in the number and coverage of PSAs for a drunk-driving prevention campaign across the U.S., offering uncertain explanations as to why such differences exist across areas, such as conflicts of interest in certain markets or market-specific competition over advertising space [19].

Given this past research as well as COVID-specific research documenting that local information available varied based on the population characteristics of communities [20], it is important to examine how the airing of COVID-19 health campaigns might also have varied based on population factors. Thus, our research aim was to understand the reach and distribution of COVID-19 PSA airings. To do so, we examined the volume and geographic distribution of federally-affiliated PSAs aired on television between March 12 and December 16, 2020. We linked data on market-level PSA airings with data on county-level COVID-19 incidence, to describe the relationship between attention to COVID-19 on television and COVID-19 cases across the nation. We also incorporated information on market-level population characteristics (age, race, education level, and partisanship) given evidence of the pandemic’s inequitable impact on communities and the role of partisanship in shaping COVID-related attitudes, behaviors, and information availability in the United States [20–24].

## Methods

### Data

We obtained data on federally-affiliated COVID-19 PSAs aired from March 12, 2020 (the earliest date ads were available) through December 16, 2020 from Kantar/CMAG. Kantar/CMAG searched their database of ads aired on local and national broadcast TV to identify all distinct ads (i.e., creatives) that were related to COVID-19 from the U.S. Department of Health and Human Services, the Ad Council, and/or Coronavirus.gov. The dataset included 222 creatives aired 235,734 times. To verify that only federally-affiliated creatives were included, three researchers viewed each of the 222 creatives and coded the sponsor of ads based on observable symbols (e.g., an Ad Council or White House logo). Inter-rater reliability for this assessment was high (Krippendorff’s alpha exceeded 0.97). We restricted our analytic sample to 132 creatives that were aired 170,820 times during the study period and were sponsored by the Ad Council, CDC, CDC Foundation, Federal Emergency Management Agency, U.S. Department of Health and Human Services, or the White House. Almost all (99.5%) of the airings included Ad Council branding, often alongside the other federal designations. In a previous study, we examined the health informational content of these ads, including celebrities depicted, the major health messages broadcast, and attention to groups at risk [25].

The Kantar data included information about the media market (i.e., designated market area, or DMA), date, station, and time each ad was aired. We used a county-to-DMA crosswalk file constructed from public sources to merge the PSA data with county-level COVID-19 cases from the publicly available *New York Times* repository of COVID-19 data [26]. We constructed two measures of COVID-19 incidence at the market level: (1) the seven-day rolling average of daily COVID-19 cases; and (2) the total number of COVID-19 cases over two periods, the “first wave” (March 12, 2020 –June 9, 2020) and the “second wave” (June 10, 2020 –December 16, 2020). Waves are distinguished at June 9, 2020, when U.S. cases hit 2 million and the declining seven-day average of new cases first flattened before subsequently increasing. We converted both measures to cases per 100,000 population.

We also merged other characteristics reported at the county-level, using the same crosswalk file to aggregate counties into media markets. We included the share that voted for the Democratic candidate, Hillary Clinton, in the 2016 Presidential election [27], the measure a recent study used to examine the relationship between partisanship and local COVID information [20]. We also incorporated data on the market population size, the percent of the population under 18 years old, the percent of the population over 65 years old, the percent with some college or more education, and the percent of the population that identified as people of color (non-white), all from the American Communities Study. These factors have been associated with county-level COVID-19 impact [23, 24].

### Statistical analysis

To estimate whether the volume of ad airings in a community was associated with characteristics of the market population, we estimated generalized linear regression models predicting the count of PSA airings as a function of COVID-19 case rates and the other characteristics described above, for the whole period and within the two waves. We specified a negative binomial distribution for the outcome variable and used a log link function.

## Results

**Fig 1:**
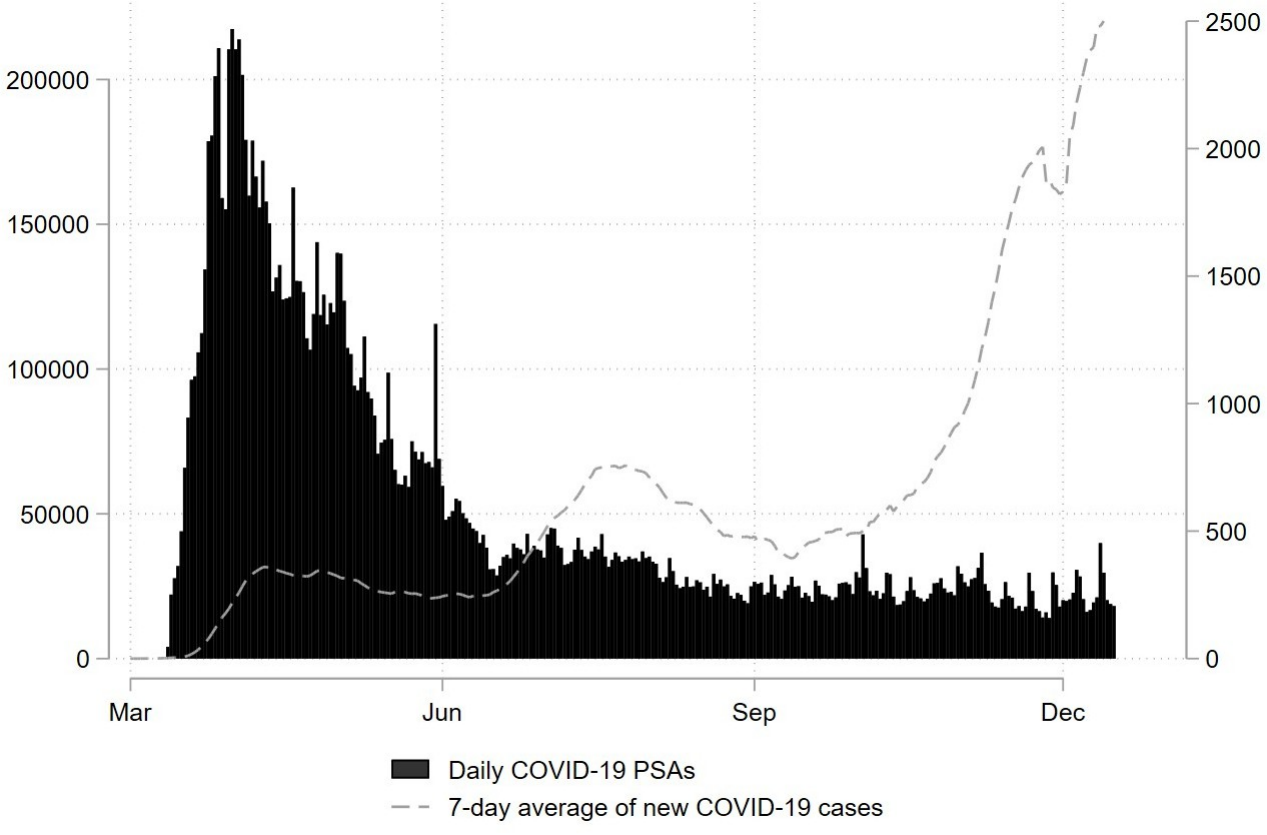
Daily federal PSA Ad airings and COVID-19 cases in the United States, March to December 2020. Authors’ analysis of data of PSA airings from Kantar/CMAG merged with data on COVID-19 incidence from the New York Times.

The analytic sample included 132 distinct creatives (i.e., ads) that were aired 170,820 times during the study period. Fig 1 displays the volume of PSA airings over time, plotted against the seven-day average of new COVID-19 cases. While a high volume of federal PSAs aired during the first months of the pandemic (March through May), airings did not track the escalating severity, and far fewer ads were aired from June to December.

We also examined the distribution of the timing of ad airings. Only 6.3% of airings were during “prime time” programming (typically 8-11pm Eastern), the most desirable block due to audience size. Most were aired during the early morning, typically 6am to 9am (28.5%) or during daytime programming, typically 9am to 4:30pm (21.5%); a substantial proportion were aired during late night programming (23.9%).

**Fig 2:**
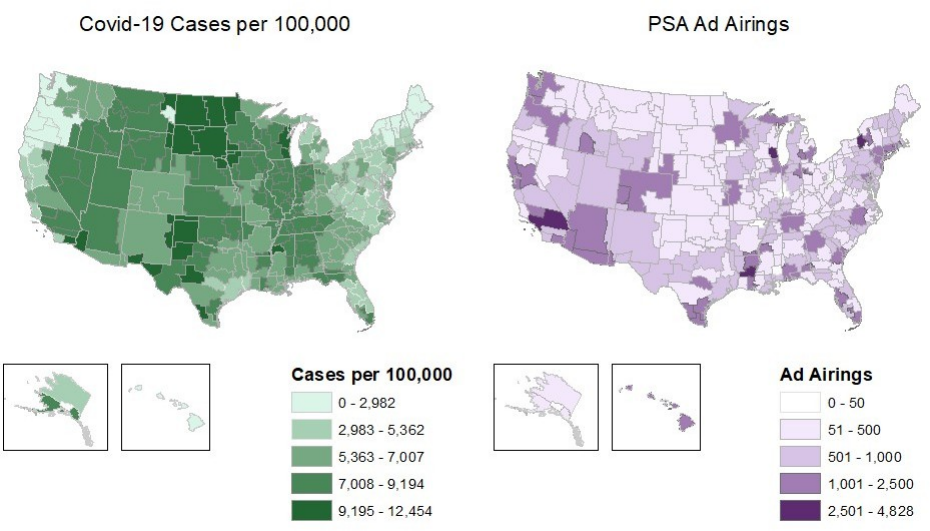
Maps of COVID-19 cases and PSA Ad airings, by media market in the U.S., March-December 2020. Authors’ analysis of data of PSA airings from Kantar/CMAG and data on COVID-19 incidence from the New York Times.

Next, we examined geographic variation in airings (Fig 2). The left-hand panel displays COVID-19 cases per 100,000 population at the media-market level, where darker colors indicate higher COVID-19 incidence. The right-hand panel displays the volume of PSAs aired in each media market, where darker colors indicate more PSAs aired. Whereas the highest rates of COVID-19 were in markets in North and South Dakota, as well as some in Arizona and Texas, the highest volume of PSA airings were in markets in upstate New York (Syracuse), northern Virginia (Alexandria), Wisconsin (Milwaukee) and Southern California (Los Angeles).

Table 1 examines the associations between PSA airings, COVID-19 case rates, and other characteristics. Media markets where the population voted in greater shares for Clinton in 2016 saw more exposure to PSA airings in 2020, in both pandemic waves. During the first wave, each additional percentage point of Clinton vote share was associated with about 12.5 additional PSAs airing in the market; this relationship attenuated to 7.0 PSAs during the second wave. More populous markets also saw more PSAs aired, 4.0 airings per additional 100,000 population during the period. The share of the market with some college was negatively correlated with PSA airings over the whole study period. Across all three models, there was no statistical relationship identified between the COVID-19 case rates in a market, nor other demographic characteristics of the market, and the volume of PSAs aired there.

**Table 1:** Association of PSA airings and market-level factors, overall and by pandemic wave.

	Full Time Period		Wave 1		Wave 2	
	Coeff (SE)	p-value	Coeff (SE)	p-value	Coeff (SE)	p-value
Cases per 100k	0.00 (0.02)	0.957	0.01 (0.07)	0.847	–0.01 (0.01)	0.452
Vote share for Clinton in 2016	20.5 (6.4)	0.001	12.5 (3.9)	0.001	7.0 (2.9)	0.016
Proportion non-white	–4.2 (4.1)	0.303	-2.1 (2.5)	0.398	–1.7 (2.0)	0.378
Proportion age <18	–5.8 (28.5)	0.839	-7.7 (18.3)	0.675	2.5 (13.9)	0.858
Proportion age 65+	–40.8 (25.4)	0.108	-24.3 (14.7)	0.098	–16.4 (12.9)	0.206
Proportion with some college or more	–21.2 (10.2)	0.038	-9.6 (6.0)	0.107	–10.4 (4.9)	0.032
Market population (100k)	4.0 (1.6)	0.013	3.5 (1.2)	0.003	0.4 (0.7)	0.571
*N*	209		209		209	

Notes. Authors’ analysis of data of PSA airings from Kantar/CMAG merged with data on COVID-19 incidence from the New York Times and market-level demographic data constructed from data from the Atlas of Presidential Elections and the American Community Survey. Wave 1 refers to March 12 –June 9, 2020; Wave 2 refers to June 10 –December 16, 2020. Coefficients and standard errors are the change in the count of PSAs associated with a one unit change in the independent variable. Results come from a DMA-level generalized linear model with a negative binomial distribution and log link. Robust standard errors in parentheses.

**Fig 3:**
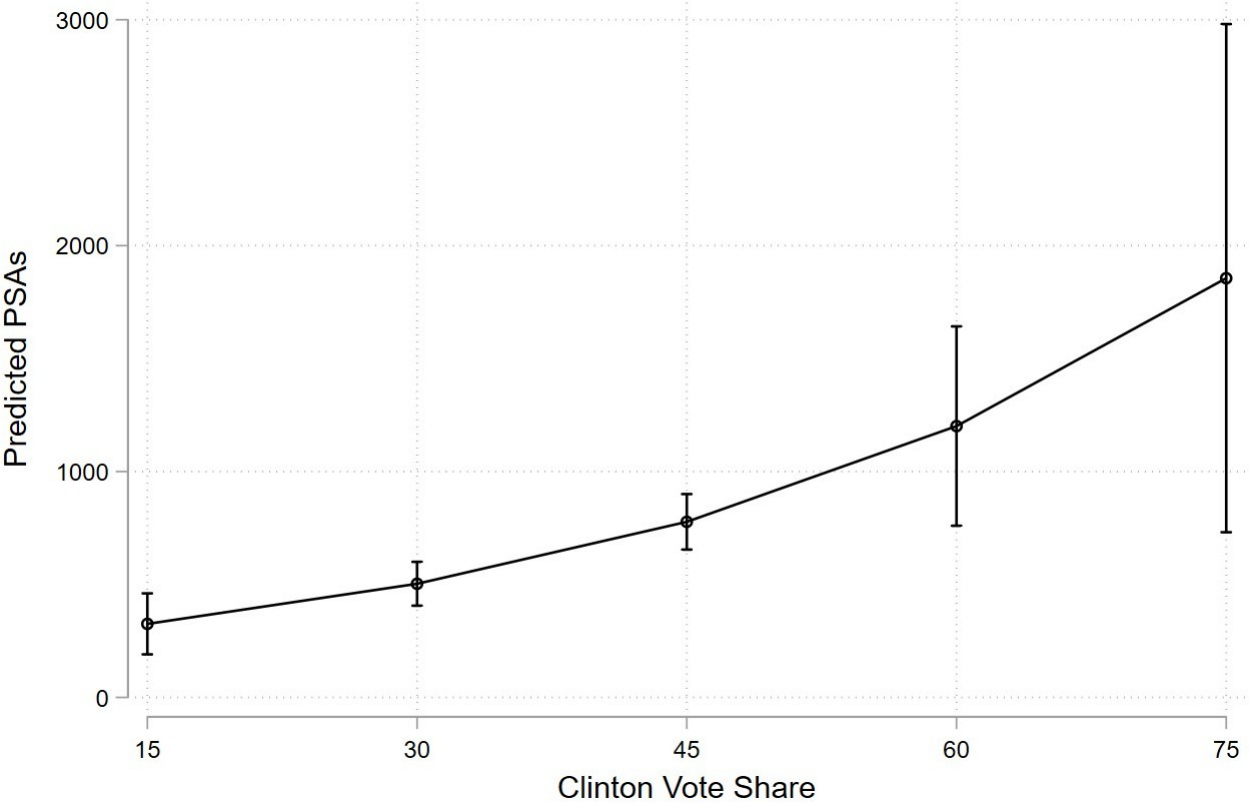
Predicted PSA volume by Clinton vote share. Authors’ analysis of data of PSA airings from Kantar/CMAG merged with data on COVID-19 incidence from the New York Times and market-level demographic data constructed from data from the Atlas of Presidential Elections and the American Community Survey. Figure displays the predicted PSA volume based on the full time period regression model displayed in Table 1, by Clinton vote share, adjusting for all other characteristics listed in Table 1.

Fig 3 uses the results in Table 1 to display how the predicted number of PSAs changes with the Clinton vote share. Not only did the predicted number of PSAs increase as the Clinton vote share increased, but the slope of these changes was also larger as the Clinton vote share grew. Moving from a 30 percent vote share to a 45 percent vote share was associated with a 274 airing increase in the predicted number of PSAs. Moving from a 60 percent vote share to a 75 percent vote share was associated with a 655 airing increase.

## Discussion

During the first ten months of the COVID-19 pandemic in the United States, 132 PSAs created as part of a federal partnership with Ad Council were aired on television more than 170,000 times. Our analysis reveals that these ads were not strategically aired to be responsive to the incidence of COVID-19. The bulk of ads were aired during the first few months of the pandemic, and they were aired during times with relatively lower TV audiences. These findings suggest that the federal government may not have optimized the use of PSAs to provide timely health information to Americans in 2020. Further, our analysis suggests that health information in PSAs was more available to people living in Democratic-leaning media markets compared to those in Republican-leaning markets. This finding contributes additional evidence of how the availability of health information about COVID-19 was unequally distributed by mass partisanship during this crucial stage of the pandemic [20].

Despite the gravity of the health emergency of the COVID-19 pandemic in 2020 and urgent need to communicate with the public, our findings related to PSA distribution and spread are similar to the limited previous research on other PSA campaigns. For example, previous studies also demonstrated that prime time was the least popular time block for airing PSAs [4, 7, 17, 19]. While we found that 6.3% of COVID-19 ads were aired during prime time, a comprehensive 2008 study by the Kaiser Family Foundation revealed that 13% of all PSAs were aired during prime time [7], suggesting COVID-19 ads were even less of a priority to air in this highest-viewed time block compared to the past. One explanation for the low airing during prime time and later in the fall could be due to crowd-out of space because of the 2020 election, since prime time ads spots are of high value to political campaigns, particularly in the fall [28, 29]. The wide geographic variation we observed across media markets in the volume of airings is consistent with previous research [19].

Our study results raise key questions about the mechanisms through which these COVID-19 ads were aired, and especially the strategic choices that contributed to these political geographic patterns in airings. Surprisingly little is known about how or why ads get aired on some stations or broadcasts over others. A comprehensive, albeit dated, review of public service communication from 1990 stated that “dissemination of any particular PSA is at the discretion of the network, station, or publication management, and content as well as stylistic and production factors can influence decisions on whether to present it, and if so, when and where” [3]. We have little reason to believe this is no longer true. As a 2013 study examining audience reach of a colorectal cancer campaign put it, “Campaign planners have little control over whether, when, where, or how often intended audiences are exposed to public service announcements” [30]. We are unable to speculate from this study what specific factors currently shape whether or not a station producer chooses to air a PSA, although review of publically-available tips and advice for non-profits producing PSAs indicates that stations still have great discretion over these decisions, suggesting these placement decisions may be idiosyncratic and based on the ad content itself as well as competitive pressures of other ads [31–33]. This is clearly an important area for future research, particularly given the politicization of health information during COVID-19. It could, for instance, be possible that stations with decision-makers who are less supportive of the COVID-19 pandemic (or who live in communities with those attitudes) may have been less likely to run COVID-19 PSAs generally, or those with particular messages (e.g., recommending masking, etc.) While our results do not indicate the specific reasons why exposure to health information was patterned politically, this distribution of important health information could have potentially contributed to the divergent COVID-19 attitudes, beliefs, and behaviors observed across Republicans and Democrats in 2020 (and sustained into 2021 and beyond) [34–36].

### Limitations

Our data have some limitations. First, Kantar/CMAG includes all national broadcast, cable, and local television but not local cable advertisements. Second, the Kantar system to capture new creatives includes only a subset of media markets (133 of the full 210 in 2020, including at least 1 market in every state), although they track the airings of every creative captured across all 210 media markets [37]. This means that an ad that aired exclusively in a smaller-population market would not appear in the data set, although this is unlikely an issue given our focus on federally-affiliated ads (i.e., not those created by local agencies). Third, PSAs related to COVID-19 were created by many entities in 2020, from local organizations to state health departments to celebrities sharing on social media [18]. Our data do not include all the PSAs to which Americans were exposed during this time, such as on radio, streaming services, social media, or billboards. Further, some PSAs created by Ad Council (based on examining CDC and Ad Council websites) were not available in our dataset, suggesting they may have been aired only digitally (i.e., YouTube) or exclusively on local cable television. Despite these limitations, we believe this to be the most comprehensive available dataset tracking the airing of federally-affiliated COVID-19 PSAs.

## Conclusions

Investment in communication campaigns is one important policy strategy that shapes the public’s attitudes, beliefs, and behaviors about COVID-19. Our findings indicate that this strategy, among ads identified as being affiliated with the federal government’s response, was not necessarily responsive to the changing COVID-19 case rates over 2020. Such an approach might have been in the interest of communicating information with urgency and thus less responsive to the evidence-base of how and where to communicate for behavior change [18, 38]. While this descriptive analysis offers insights into one aspect of the health communication that reached the public in 2020, questions remain. For example, as noted previously, it is unknown (to this study team) how stations selected into airing, or not airing, the PSAs made available through the federal partnership and how costs may factor in these decisions [8]. Additional research might examine station-level factors that relate to airing ads or not (e.g., station ownership) as well as ascertaining more detail on the process through which the specific creatives were selected to be aired, such as through qualitative research. The 2008 Kaiser Family Foundation study referenced earlier, for instance, used letters, emails, and phone calls to stations, cable networks, and ad sponsors to understand more about how over 1,500 PSAs aired in fall 2005 were paid for, a huge undertaking [7]. Future research might also continue to track PSAs aired in 2021 and beyond, to examine the content of the specific health messages communicated by the PSAs once the COVID-19 vaccines were available. Overall, the PSAs in this study were just one component of the overall COVID-19 health communication environment that influenced the public’s understanding and response in 2020. More research is needed to examine how health messaging, whether the federal investment in strategic messaging like PSAs or the incidental health messaging available in news coverage, shaped Americans’ COVID-related attitudes and behaviors, in 2020 and beyond.
